# A Study of Correlation of Various Growth Indicators with Chronological Age

**DOI:** 10.5005/jp-journals-10005-1311

**Published:** 2015-09-11

**Authors:** Sarabjeet Singh, Navreet Sandhu, Taruna Puri, Ritika Gulati, Rita Kashyap

**Affiliations:** Professor and Head, Department of Orthodontics, Bhojia Dental College, Baddi Himachal Pradesh, India; Associate Professor, Department of Prosthodontics, Bhojia Dental College, Baddi Himachal Pradesh, India; Senior Lecturer, Department of Orthodontics, Bhojia Dental College, Baddi Himachal Pradesh, India; Resident, Department of Orthodontics, Bhojia Dental College, Baddi Himachal Pradesh, India; Reader, Department of Orthodontics, Bhojia Dental College, Baddi Himachal Pradesh, India

**Keywords:** Growth indicators, Cervical vertebrae maturation index (CVMI), Frontal sinus width, Antegonial notch depth.

## Abstract

**Introduction:** The aim of this study was to assess the relationship of chronological age with cervical vertebrae skeletal maturation, frontal sinus width and antegonial notch depth and a correlation, if any, among the three variables.

**Materials and methods:** The samples were derived from lateral cephalometric radiographs of 80 subjects (40 males, 40 females; age range: 10 to 19 years). Cervical vertebral development was evaluated by the method of Hassel and Farman, frontal sinus width was measured by the method described by Ertürk and antegonial notch depth as described by Singer et al. The Pearson’s correlation coefficients were estimated to assess the relationship of chronological age with cervical vertebrae skeletal maturation, frontal sinus width and antegonial notch depth.

**Results:** The Pearson’s correlation coefficient were 0.855 (p < 0.001) between chronological age and cervical vertebrae skeletal maturation, and 0.333 (p < 0.001) between chronological age and frontal sinus width.

**Conclusion:** A highly significant positive correlation was found between chronological age and cervical vertebrae skeletal maturation, and between chronological age and frontal sinus width. Nonsignificant correlation was found between chronological age and antegonial notch depth.

**How to cite this article:** Singh S, Sandhu N, Puri T, Gulati R, Kashyap R. A Study of Correlation of Various Growth Indicators with Chronological Age. Int J Clin Pediatr Dent 2015;8(3): 190-195.

## INTRODUCTION

The ability to predict the magnitude and direction of patient’s facial growth early in life would enable the clinician to identify those who require interceptive growth modification, and to ensure that the appropriate treatment can be rendered while growth is possible. Several clinical and animal experimental studies have shown the significant role played by the function of muscles of mastication in craniofacial growth.^1,2^ In addition, the ability to predict facial growth might enable the clinician to forego unnecessary treatment on patients with skeletal discrepancies, whose growth pattern would probably lead to correction without orthopedic intervention. Therefore, a reliable method of growth prediction would be an invaluable asset to orthodontists.^3^

The maturational status can also have considerable influence on the diagnosis, treatment planning and eventual outcome of orthodontic treatment.^4^ Clinical decisions regarding the use of extraoral traction force, functional appliances, extraction *vs* non-extraction treatment or orthognathic surgery are mainly based on growth considerations. The various maturity indices that have been used to identify stages of growth are—sexual maturation characteristics, chronological age, dental development, height, weight and skeletal development.^5^

Hassel and Farman utilized the cervical vertebrae and found them as reliable and valid for assessing skeletal age.^3^ Frontal sinus development has also been found to show a growth rhythm similar to body height development, with a well-defined pubertal peak.^6^ Mandibular antegonial notch morphology, predicting mandibular growth, has also been referred in various studies.^7,8^

Hence, keeping the above mentioned growth indicators in mind, the following study was carried out to assess the relationship of cervical vertebrae maturation stages, frontal sinus width and antegonial notch depth with chronological age; and to assess the correlation, if any, among all the above parameters.

## MATERIALS AND METHODS

The present study was conducted on 80 north Indian children (40 males, 40 females, age range of 10 to 19 years). All the subjects selected were moderately built and in growing age with no history of bone deformities, bone diseases and major illness in the past.

A written consent was obtained from the parents of selected subjects, after explaining the nature of the study.

For all subjects, a standard digital lateral cephalogram was taken in a natural head position using the machine Advapex (OPG-TMJ-CEPH, flat cassette model); to evaluate cervical vertebrae maturation stage, frontal sinus width and antegonial notch depth.

Radiographs of high clarity and good contrast with standardized processing technique were used, and interpretation of all radiographs was undertaken without referring to age of patient, to avoid selection bias.

**Fig. 1:**
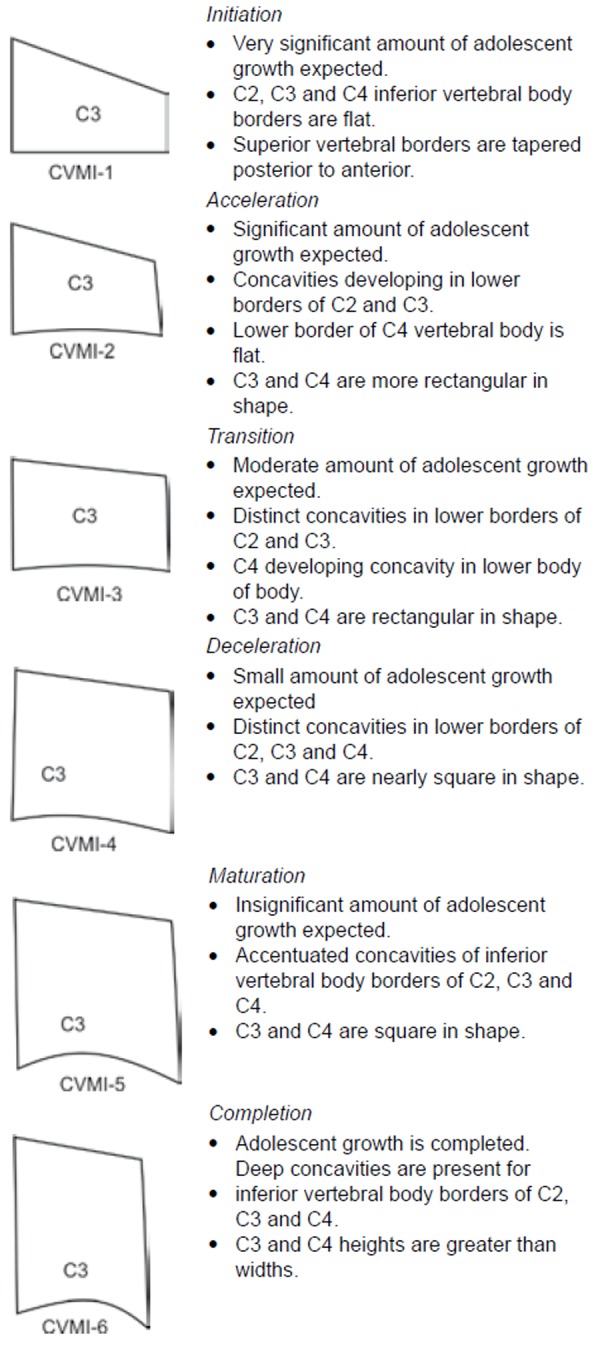
Cervical vertebrae maturation index stages

Cervical vertebrae stages were determined by the Hassel and Farman modification of the criteria of Lamparski (Fig. 1).^5^ The cervical vertebrae maturation stages were rated by two orthodontists separately, and without knowing chronologic ages. The average of these ratings was used as the vertebrae maturation stage.

**Fig. 2:**
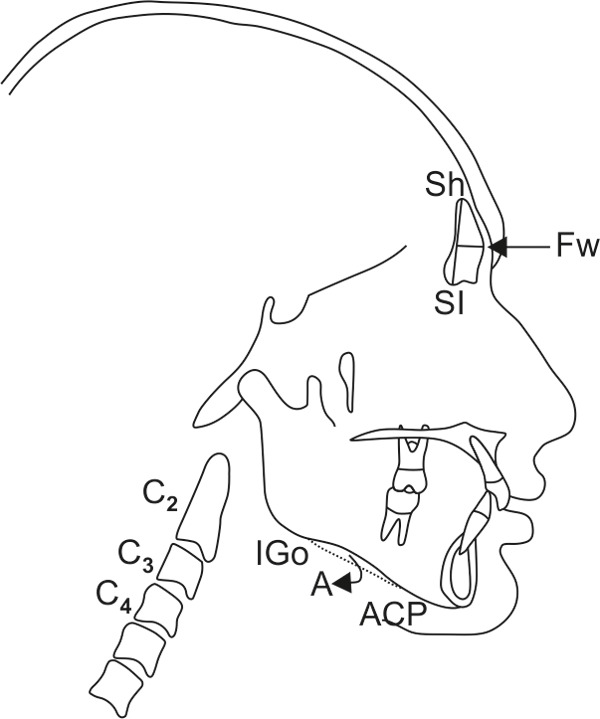
Cervical vertebrae maturation index stages Hassel and Farman (1995), frontal sinus width (Ertürk, 1968), antegonial notch depth (Singer et al, 1987)

Width of the frontal sinus was measured by the method described by Ertürk (1968)^9^ in which the lateral head films were orientated with the nasion-sella line horizontally. The peripheral border of the frontal sinus was traced and the highest (Sh) and lowest (Sl) point of its extension were marked. Perpendicular to the interconnecting line Sh-Sl, the maximal width of the frontal sinus was assessed (Fig. 2).^10^

Mandibular antegonial notch depth was measured by method described by Singer et al, that included landmarks anterior convexity point (ACP) and inferior gonion (Igo).^11^ The depth was measured from greatest point of convexity in antegonial notch to line connecting ACP with Igo along a line perpendicular to ACP-Igo line (Fig. 2). Chronological age was verified by an appropriate authentic document like birth certificate, Aadhaar card, etc. All the above parameters were assessed on each subject and comparison was done.

## STATISTICAL ANALYSIS

The means, standard deviations, and standard errors were calculated for each parameter with a software package (SPSS for Windows 98, version 10.0, SPSS, Illinois, Chicago, USA). The significance of difference between mean values was evaluated by the unpaired t-test. Differences were considered statistically significant when the ‘p’ value was 0.05 or less. Pearson’s correlation coefficients were calculated between chronological age with Cervical vertebrae maturation index (CVMI), frontal sinus width and antegonial notch depth, and between the above three variables.

## RESULTS

Table 1 shows the number and the respective mean ages of male and female patients. The patients were matched for age and sex by t-test; there was no significant difference between the mean age of male and female patients.

The gender-wise distribution of number of subjects within each CVMI stages are shown in Table 2. The most frequent cervical vertebra stages were stage 2 (25%) and stage 3 (22.5%) in males; and stage 3 (30%) and stage 5 (22.5%) in females.

Table 3 shows the mean values of frontal sinus width and antegonial notch depth in the male and female subjects. The difference between the frontal sinus width and antegonial notch depth among female and male subjects were compared using unpaired t-test and a statistically significant (p = 0.006) difference between the male and female subjects was noted in frontal sinus width, while there was no difference in the antegonial notch depth (Table 4).

Table 5 shows the age, frontal sinus width and antegonial notch depth among male and female subjects, divided according to the three age-groups of 10 to 12, 13 to 15 and 16 to 19 years, respectively. There was no difference between mean age, frontal sinus width and antegonial notch depth among male and female subjects in all the age groups, except the frontal sinus width in the 13 to 15 years age group where the difference was statistically significant (p = 0.002) (Table 6).

A Pearson’s correlation analysis was done to find the correlation between age and CVMI, frontal sinus width and antegonial notch depth, and among each other. The results are shown in the Tables 7 to 10.

We observed a significant positive correlation of age with CVMI, and age with frontal sinus width, and vice versa. A nonsignificant correlation was found between chronological age and antegonial notch depth.

The various statistical data are also represented in graphs in Graphs 1 to 8.

**Table 1:** **Table 1:** Mean age of male and female subjects in the study

*Group*		*Number*		*Mean age (years)*	
Male		40		13.85 ± 2.28	
Female		40		13.70 ± 1.88	
Total		80		13.78 ± 2.08	

**Table 2:** **Table 2:** The distribution of male and female subjects according to the various CVMI stages

*CVMI Stage*		*Number of male subjects (n = 40)*		*Number of female subjects (n = 40)*		*Total number of subjects (n = 80)*	
1		5 (12.5%)		0 (0%)		5 (6.3%)	
2		10 (25.0%)		7 (17.5%)		17 (21.3%)	
3		9 (22.5%)		12 (30.0%)		21 (26.3%)	
4		6 (15.0%)		7 (17.5%)		13 (16.3%)	
5		7 (17.0%)		9 (22.5%)		16 (20.0%)	
6		3 (7.5%)		5 (12.5%)		8 (10.0%)	

## DISCUSSION

In orthodontics and dentofacial orthopedics, the assessment of skeletal age and pubertal growth spurt, in particular, are of prime importance in diagnosis, treatment planning and retention after orthodontic treatment. It is important to identify the individual maturational levels of a child to evaluate expected developmental events. Stages of maturation can be determined, or at least estimated in several ways.^3^

It is necessary for an orthodontist to know the leftover growth potential during the period of treatment and the percentage of growth expected at the time of treatment. Conventional prediction indicators of maturation overestimate a child’s developmental stage, and consequently, underestimate growth potential. Racial variations between skeletal maturity established by various methods have been previously reported. For this reason, this study was carried out to investigate the relationships among chronological age with cervical vertebrae maturity indicators, frontal sinus width and antegonial notch depth of north Indian subjects.

As orthodontic treatment is frequently performed at the age group of 10 to 19 years, it was selected for the study. The lateral cephalograms of 80 healthy north Indian children were taken to assess the CVMI stages, frontal sinus width and antegonial notch depth. The analysis of the radiographs was carried out twice at the interval of 15 days to evaluate the intraoperator error. Since the t-test for all three variables between two measurements of same radiographs were insignificant, the correlation between two observations is highly significant, which indicates that the measurement recorded for this study is quite reliable and acceptable.

**Table 3:** **Table 3:** The mean frontal sinus width and antegonial notch depth in the male and female subjects

*Group*		*Frontal sinus width*		*Antegonial notch depth*	
Male		7.03 ± 1.48		1.31 ± 0.54	
Females		6.05 ± 1.60		1.21 ± 0.50	
Total		6.54 ± 1.61		1.26 ± 0.52	

**Table 4:** **Table 4:** Difference between frontal sinus width and antegonial notch depth among male and female subjects

*Parameters*		*Mean difference*		*Standard error*		*p-value*	
Frontal sinus width		0.98		0.35		0.006*	
Antegonial notch depth		0.10		0.12		0.395	

*p < 0.05 is considered significant

**Table 5:** **Table 5:** The age, frontal sinus width and antegonial notch depth in three different age groups among male and female subjects

		*10-12 years*				*13-15 years*				*16-19 years*			
*Parameters*		*M (n = 12)*		*F (n = 12)*		*M (n = 17)*		*F (n = 20)*		*M (n = 11)*		*F (n = 8)*	
Age		11.25 ± 0.87		11.67 ± 0.65		13.82 ± 0.88		13.75 ± 0.85		16.73 ± 1.01		16.63 ± 0.52	
Frontal sinus width		6.21 ± 1.51		5.83 ± 0.96		7.32 ± 1.24		5.80 ± 1.45		7.45 ± 1.56		7.00 ± 2.44	
Antegonial notch depth		1.46 ± 0.58		1.33 ± 0.43		1.18 ± 0.40		1.03 ± 0.35		1.36 ± 0.66		1.50 ± 0.76	

**Table 6:** **Table 6:** Difference between the various parameters of age, frontal sinus width and antegonial notch depth between the three age groups

		*Mean difference ± standlard error of difference*											
*Age groups (years)*		*Age (years)*		*p-value*		*Frontal sinus width*		*p-value*		*Antegonial notch depth*		*p-value*	
10-12		–0.42 ± 0.31		0.196		0.37 ± 0.52		0.477		0.13 ± 0.21		0.556	
13-15		0.07 ± 0.29		0.798		1.52 ± 0.45		0.002*		0.15 ± 0.12		0.231	
16-19		0.10 ± 0.39		0.797		0.45 ± 0.91		0.625		–0.14 ± 0.33		0.682	

*p < 0.05 is considered significant

**Table 7:** **Table 7:** Correlation of CVMI, frontal sinus width and antegonial notch depth with age

*Parameters*		*Pearson’s correlation coefficient*	
CVMI		0.855*	
Frontal sinus width		0.333*	
antegonial notch depth		0.000	

*significant correlation

**Table 8:** **Table 8:** Correlation of age, frontal sinus width and antegonial notch depth with CVMI

*Parameters*		*Pearson’s correlation coefficient*	
CVMI		0.855*	
Frontal sinus width		0.207	
Antegonial notch depth		0.054	

*significant correlation

**Table 9:** **Table 9:** Correlation of age, CVMI and antegonial notch depth with frontal sinus width

*Parameters*		*Pearson’s correlation coefficient*	
Age		0.333*	
CVMI		0.207	
Antegonial notch depth		0.180	

*significant correlation

**Table 10:** **Table 10:** Correlation of age, frontal sinus width and CVMI with antegonial notch depth

*Parameters*		*Pearson’s correlation coefficient*	
Age		0.000	
Frontal sinus width		0.054	
CVMI		0.180	

**Graph 1:**
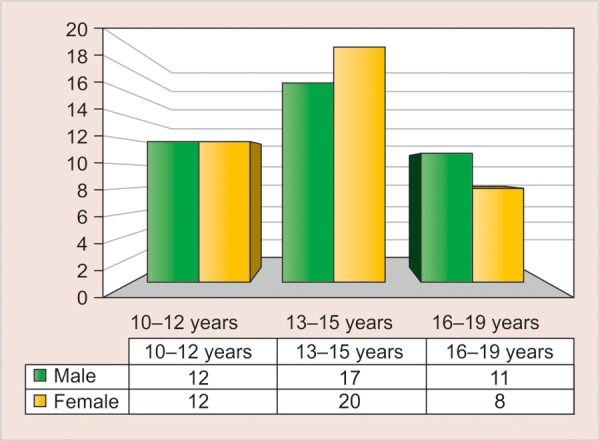
The sex distribution of subjects among the various age-groups used in the study

**Graph 2:**
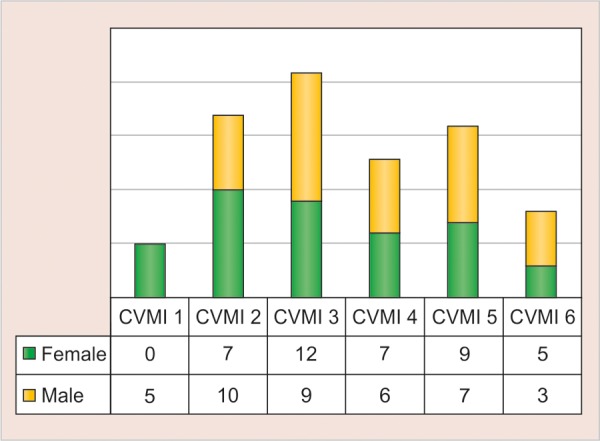
The distribution of male and female subjects in various CVMI stages

On comparing the mean age of the male and female subjects in different subgroups as shown in Table 1, it is observed that they are insignificantly (p > 0.05) different from each other. Insignificant differences in mean variation indicate that the sample used in this study is homogenous.

As the Hassel and Farman classification includes a more detailed description of every stage and uses a limited number of vertebral bodies to perform the staging, so it was used in this study.^5^

Sierra found that the relationships between chronologic age and each of the two skeletal age assessment methods (Todd inspectional method and ossification centers method) proved to have relatively high correlations, with correlation coefficients ranging from 0.58 to 0.71.^12^ In this study, the correlation between chronologic age and skeletal maturation assessed by the cervical-vertebrae was 0.855, higher than those reported by Sierra.^12^

**Graph 3:**
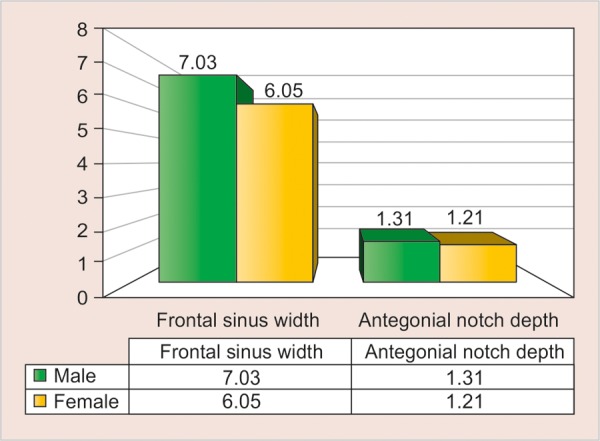
The frontal sinus width and antegonial notch depth among male and female subjects

**Graph 4:**
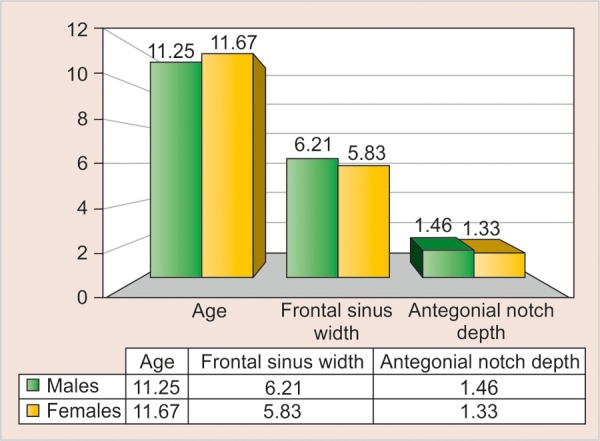
The age, frontal sinus width and antegonial notch depth in the age group 10 to 12 years

**Graph 5:**
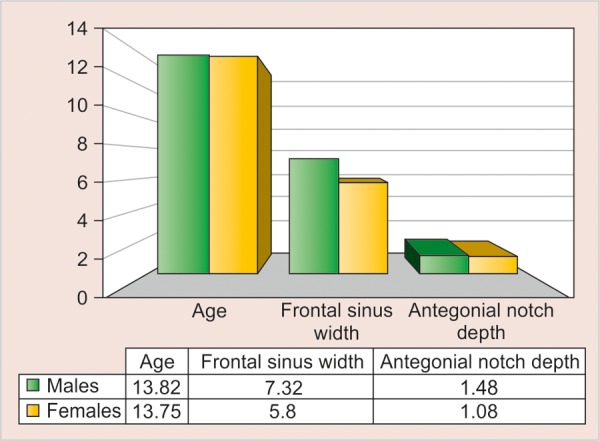
The age, frontal sinus width and antegonial notch depth in the age group 13 to 15 years

**Graph 6:**
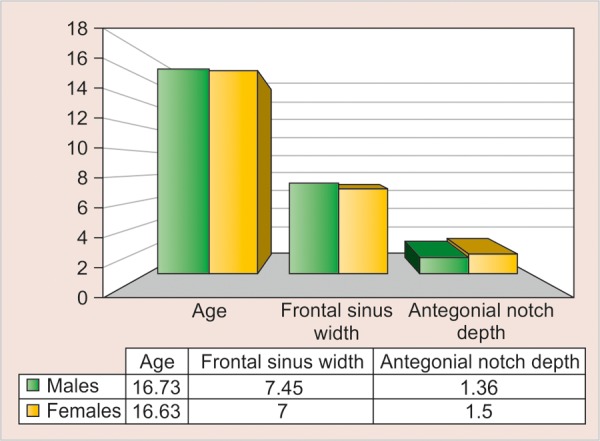
The age, frontal sinus width and antegonial notch depth in the age group 16 to 19 years

**Graph 7:**
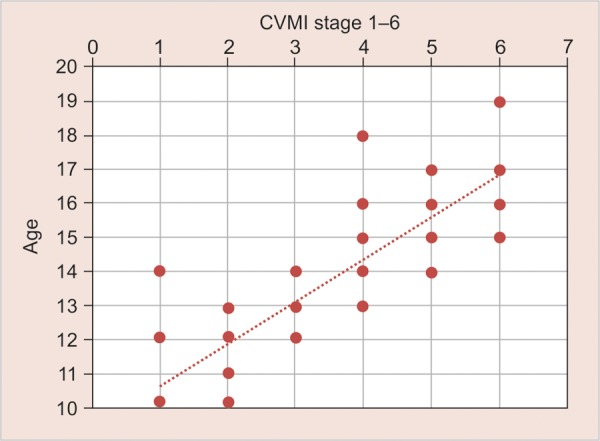
Correlation of CVMI stages with age (correlation coefficient—0.855)

**Graph 8:**
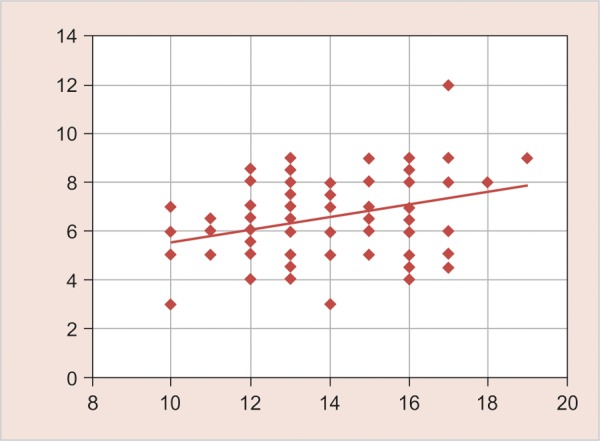
Correlation of frontal sinus width with age (Correlation coefficient—0.333

Rossouw et al (1991) correlated frontal sinus area with excessive mandibular growth.^13^ Ruf and Pancherz (1996) suggested that the somatic maturity stage may be predicted rather accurately by analyzing frontal sinus development on pre-existing lateral head cephalograms.^6^ Ruf and Pancherz (1996) reported that frontal sinus shows peak growth at an average age of 15.1 years a in males.^6^ According to Brownet al (1984) enlargement of the frontal sinus ceases at 15 years and 6 months in boys and 13 years and 9 months in girls.^14^

The results of this study show that there is a significant correlation of frontal sinus width with age, and was found to be 0.333; though there was an inconsistent correlation of frontal sinus width with CVMI as shown in Table 9.

In recent years, some researchers have proposed that the depth of the mandibular antegonial notch can be used as a predictor of facial growth. These studies were based on the findings of Bjork,^7^ who reported that mandibles with a forward growth tendency exhibit a pattern of surface apposition below the symphysis and surface resorp-tion under the mandibular angle. The opposite pattern occurred in persons with a backward mandibular growth tendency, leading to concavity on the inferior border of the mandible, known as the antegonial notch. The results of this study show that there is an insignificant correlation between antegonial notch depth and chronological age, and also between antegonial notch depth with frontal sinus width and CVMI.

It was found that at the same chronological age, there was highly significant difference (p < 0.01) in each of the three subgroups with CVMI stages, more advanced in females. Similar type of sexual dimorphism regarding the maturational parameters has been earlier reported by Joseph (1951),^15^ Hunter (1966),^16^ Fishman (1982)^17^ and Hagg and Taranger (1982).^18^

Difference between frontal sinus width among male and female subjects was highly significant, while that of antegonial notch depth was nonsignificant.

The findings in this study reveal that there is highly significant correlation between chronological age and CVMI, and chronological age and frontal sinus width, but there is a nonsignificant correlation between chronological age and antegonial notch depth.

## CONCLUSION

In determining the relationships among chronological age, cervical vertebrae, frontal sinus width and antegonial notch depth of 80 north Indian subjects, the following general conclusion can be drawn:

- The most frequent cervical vertebra stages were 2 (25%) and 3 (22.5%) in males, and 3 (30%) and 5 (22.5%) in females.
- A highly significant positive correlation was found between chronological age and cervical vertebrae skeletal maturation.
- A significant positive correlation was also found between chronological age and frontal sinus width.
- A nonsignificant correlation was found between chronological age and antegonial notch depth.
